# Electrochemical micro analytical device interfaced with portable potentiostat for rapid detection of chlorpyrifos using acetylcholinesterase conjugated metal organic framework using Internet of things

**DOI:** 10.1038/s41598-019-56510-y

**Published:** 2019-12-27

**Authors:** Shalini Nagabooshanam, Souradeep Roy, Ashish Mathur, Irani Mukherjee, Satheesh Krishnamurthy, Lalit M. Bharadwaj

**Affiliations:** 1 Amity Institute of Nanotechnology, Amity University, Noida, Sector 125, Uttar Pradesh 201301 India; 2 Division of Agricultural Chemicals, Indian Agricultural Research Institute, New Delhi, 110012 India; 3 Nanoscale Energy and Surface Engineering, School of Engineering and Innovation, The Open University, Walton Hall Campus, Milton Keynes, MK7 6AA United Kingdom

**Keywords:** Pollution remediation, Diagnostic markers

## Abstract

An Electrochemical micro Analytical Device (EµAD) was fabricated for sensitive detection of organophosphate pesticide chlorpyrifos in the food chain. Gold microelectrode (µE) modified with Zinc based Metal Organic Framework (MOF-Basolite Z1200) and Acetylcholinesterase (AChE) enzyme served as an excellent electro-analytical transducer for the detection of chlorpyrifos. Electrochemical techniques such as Cyclic Voltammetry (CV), Electrochemical Impedance Spectroscopy (EIS) and Differential Pulse Voltammetry (DPV) were performed for electrochemical analysis of the developed EµAD. The sensor needs only 2 µL of the analyte and it was tested within the linear range of 10 to 100 ng/L. The developed EµAD’s limit of detection (LoD) and sensitivity is 6 ng/L and 0.598 µ A/ng L^−1^/mm^2^ respectively. The applicability of the device for the detection of chlorpyrifos from the real vegetable sample was also tested within the range specified. The fabricated sensor showed good stability with a shelf-life of 20 days. The EµAD’s response time is of 50 s, including an incubation time of 20 s. The developed EµAD was also integrated with commercially available low-cost, handheld potentiostat (k-Stat) using Bluetooth and the results were comparable with a standard electrochemical workstation.

## Introduction

Over the recent years, there is a growing concern of using organophosphate (OP) pesticides such as chlorpyrifos, parathion, methyl parathion, ethion, malathion in agriculture due its related environmental issues, health issues and food security^1^. For the past decade, the use of OPs in different agricultural practices has momentously increased. Therefore, it has become imperative to monitor their concentration levels for the protection of ecological systems and food supplies. These toxic substances may enter into human and animal bodies from various sources in the food chain and can cause serious health hazards such as nerve disorders, respiratory diseases, abnormal cell growth, etc.^2^, OP pesticide poisoning occurs because of the inhibition mechanism of Acetylcholinesterase (AChE) enzyme. AChE enzyme helps in the regulation of acetylcholine levels in the body^3^. These inhibitors bind to the esteratic subsite of the AChE enzyme active site causing an irreversible inhibition mechanism^4^. There are various analytical techniques available to detect these inhibiting agents such as High-Performance Liquid Chromatography (HPLC), Gas Chromatography (GC), Mass Spectroscopy (MS)^5^. These methodologies are time-consuming, expensive, and require an analytical laboratory with skilled manpower for its operation. These constraints have paved way for the development of an on-field bio-sensing platform capable of monitoring pesticide levels with excellent sensitivity.

Researchers have been working for the development of biosensor platforms based optical^6–8^, electrochemical^9–11^, piezoelectric^12^ properties. Electrochemical bio-sensors are advantageous when compared to currently available methods for OP detection, due to their low detection limit, rapid response, portability, and affordability^13^. Electrochemical transduction involves monitoring and measurement of redox (reduction-oxidation) reactions occurring at the surface of the electrode, which associates the transport of electrons between the members of the redox species for a particular value of applied potential^14,15^.

There are various types of electrodes reported in literature such as Indium Tin Oxide (ITO)^16^, Fluorine-doped Tin Oxide (FTO)^17^, Glassy Carbon electrodes (GCE)^18^, and Screen Printed electrodes (SPEs)^19^ for electrochemical sensing of OPs. These electrodes have various disadvantages from a commercialization point of view like high cost, large sample volume requirement, surface imperfections, electrode fouling, short shelf-life etc. Carbon and noble metals based electrodes show some promising results with easier fabrication techniques^20^. The reusability factor in conjunction with low fabrication costs and accurate reproducible measurements makes gold microelectrodes (µE) the ideal choice.

Recently, various nanoparticles, functional nanomaterials, nanoporous materials, nanocomposites etc have been used as transducing elements in enzyme-based electrochemical biosensors to detect OPs. For example, a platinum working electrode was modified with ZnO nanoparticles in order to detect carbosulfan^21^, poly-(amidoamine) with cystamine core immobilized with AChE enzyme was developed for the detection of carbaryl^22^ and nanocomposite composed of multi-walled carbon nanotubes, tin oxide, and chitosan constructed to detect chlorpyrifos^23^. Metal Organic Frameworks (MOFs) are currently being explored for their potential use as a transducing element for ultra-sensitive detection of OPs. MOFs can be classified based on various metals ions and their bridging ligands. Recently, the design and synthesis of zinc-based MOFs have been on the rise due to its widespread applications in sensing, gas storage, separation and catalysis owing to their captivating framework architectures and topologies^24–27^. MOFs are primarily employed for sensing applications because of its high surface area, tunable porosity and modifiable host-guest chemistry compared to traditional porous materials like zeolites, and activated carbon^28,29^. MOFs have also been explored as a bio-compatible matrix in bio-sensing applications. They act as carriers for the enzyme and gives a protective bio-compatible environment which helps in long term stability of the enzymes^30^. Fusion of the enzyme and the MOF results in the novel functional material which provides a robust substrate for both qualitative and quantitative measurement of pesticides.

In this work, a novel EµAD was developed using conjugated MOF with µEs assembled on a microfluidic platform for the detection of chlorpyrifos. MOFs were exploited as a matrix element to fabricate EµAD conjugated with the bio-recognition element (AChE enzyme) in order to facilitate the specificity and sensitivity of the sensor. The applicability of the sensor in the detection of real vegetable samples had also been investigated. The present work is the integration of EµAD with a portable potentiostat (k-Stat) having Bluetooth interface, which facilitated the overall device miniaturization for the detection of OPs in real time. Low sample volume requirement, portability, ease-of-use and response time is less than a minute, makes it an ideal candidate for potential commercialization.

## Results and Discussion

### Fabrication of EµAD

**Figure 1:**
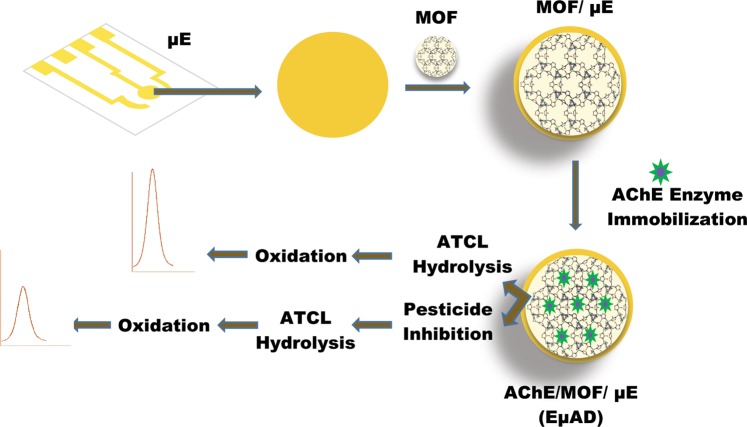
Schematic of the step-wise electrode (EµAD) fabrication for the detection of chlorpyrifos. The system includes the gold microelectrode (µE) with the all-in-one microfluidic platform (EµAD) and electrochemical analyzer for the detection of electroactive species.

As purchased gold microelectrodes (µE) were cleaned using conventional RCA-1 cleaning protocol^31^. Following the cleaning process, MOF (2% in ethanol) was drop casted on the µE and kept for drying at room temperature for 30 minutes. In the next step, AChE enzyme (in PBS, pH 7) was immobilized on the MOF/µE. These electrodes were then stored in 4 °C for further use. The schematic of the step-wise electrode (EµAD) fabrication is shown in Fig. 1. The electrodes were electrochemically examined at every fabrication step using CV and EIS as a confirmatory measure.

### Electrochemical characterization of EµAD

**Figure 2:**
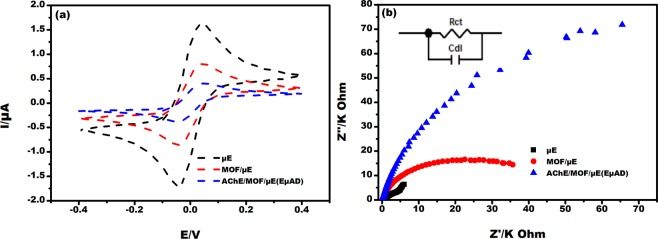
(**a**) Cyclic voltammetric studies on µE, MOF/µE, AChE/MOF/µE (EµAD) at scan rate 10 mV/s and potential −0.4 to +0.4 V in 5 mM K (Fe(CN)_6_)^3−/4−^ prepared in 0.1 M PBS pH 7. (**b**) Nyquist plot along with Randel’s equivalent circuit on µE, MOF/µE, AChE/MOF/µE (EµAD) at AC frequency 100 Hz to 100 KHz in 5 mM K (Fe(CN)_6_)^3−/4−^ prepared in 0.1 M PBS pH 7.

**Figure 3:**
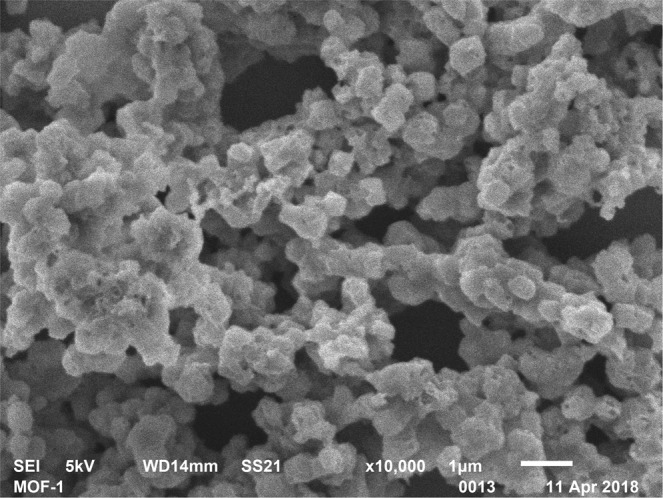
Scanning electron microscope image of as-purchased Basolite-Z1200 MOF.

**Figure 4:**
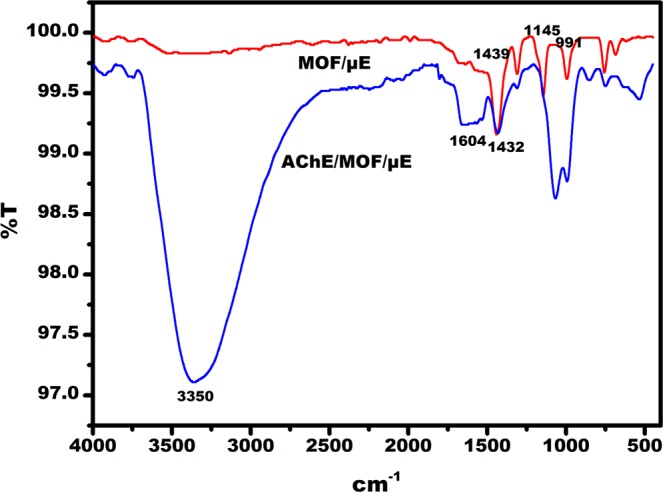
FTIR spectra of MOF/µE and AChE/MOF/µE.

Cyclic voltammogram were obtained for the µE, MOF/µE and AChE/MOF/µE (EµAD). The gold µE facilitated significant peak current enhancement in the presence of redox couple, which was attributed due to the conductive and electrocatalytic property of µE. The peak current at −0.01 V decreased from 1.6 µA to 0.7 µA after casting MOF on µE. The AChE enzyme was immobilized on the MOF/µE and peak current was further decreased to 0.4 µA. This was due to the less conductive nature of the AChE enzyme as shown in Fig. 2(a)^13^. The electrochemical response of EµAD fabrication steps was further examined using EIS as shown in Fig. 2(b). It was observed that EµAD showed lower charge transfer resistance (R_ct_) values of 4 KΩ due to the good conducting nature of µE, which further enabled the electron transfer between µE and electrolyte. After casting MOF and immobilizing AChE enzyme on the µE, the R_ct_ values further increased to 35.8 KΩ and 40 KΩ respectively, which was a direct consequence of the hindrance in electron charge transfer between the modified electrode and electrolyte^24^. The results of CV and EIS correspond well with each other confirming the successful fabrication of EµAD. Scanning Electron Microscopy (SEM) was done to study the surface morphology of as purchased Basolite Z1200 MOF (Fig. 3). SEM image depicts a uniform size distribution with the rhombic dodecahedron topology. Fourier transform infrared (FTIR) spectroscopy was done at each step of modification of the electrode to confirm the successful fabrication of a sensor (Fig. 4). For the MOF/µE, the peak at 1575 cm^−1^ was due to the C=N stretching vibration of imidazole, and the bands in the range of 1350−1500 cm^−1^ were associated with the imidazole ring stretching. The strong bands at 1145 and 991 cm^−1^ were attributed to the C−N stretching of the imidazole units. In AChE/MOF/µE the band at 1604 cm^−1^ represents NH bending and scissoring mode of AChE enzyme. At 1432 cm^−1^ the peak was attributed to C = C stretching vibrations. The peak around 3350 cm^−1^ is due to the presence of free hydroxyl stretching mode in AChE enzyme.

### Optimization of sensor performance

**Figure 5:**
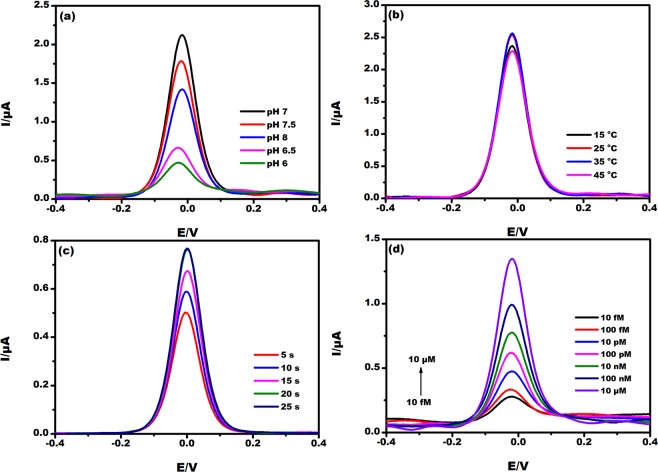
The DPV response of EµAD at (**a**) Various pH values (6, 6.5, 7, 7.5, 8) in 5 mM K [Fe(CN)_6_]^3−/4−^ prepared in 0.1 M PBS pH 7. **(b)** Different temperatures (15 °C, 25 °C, 35 °C, 40 °C) in 5 mM K [Fe(CN)_6_]^3−/4−^ prepared in 0.1 M PBS pH 7. **(c)** Various incubation time in seconds (5, 10, 15, 20, 25) in 5 mM K [Fe(CN)_6_]^3−/4−^ and 10 µM ATCl prepared in 0.1 M PBS pH 7. **(d)** Various concentrations of substrate (ATCl) ranging 10 fM to 10 µM in 5 mM K [Fe(CN)_6_]^3−/4−^ prepared in 0.1 M PBS pH 7.

The fabricated EµAD was optimized with various parameters such as incubation time, pH, temperature and substrate concentration (ATCl). DPV curve (Fig. 5(a)) depicts that at lower pH the current value increases, a maximum current of 2.1 µA was observed at pH 7. Further increase in pH decreases the peak current value, as lower pH has more H^+^ which dominates the current response. Therefore, further experiments were carried out at pH 7. Figure 5(b) shows the effect of electrolyte temperature on EµAD. The results showed no significant change in current values at temperatures ranging from 25 °C to 45 °C. The current response may decrease at elevated temperatures beyond 45 °C owing to enzyme instability at high temperatures. All further experiments were performed at an ambient temperature of 25 °C. However, the sensor can provide an effective response up to 45 °C which makes it suitable for on-field detection.

Figure 5(c) shows the effect of EµAD at different incubation time. The incubation time is the time taken for the reaction to complete once the analyte is added onto EµAD. From the DPV curve, it was observed no significant change in current response after 20 s. Therefore, the incubation time of 20 s was fixed for the subsequent experiments.

**Figure 6:**
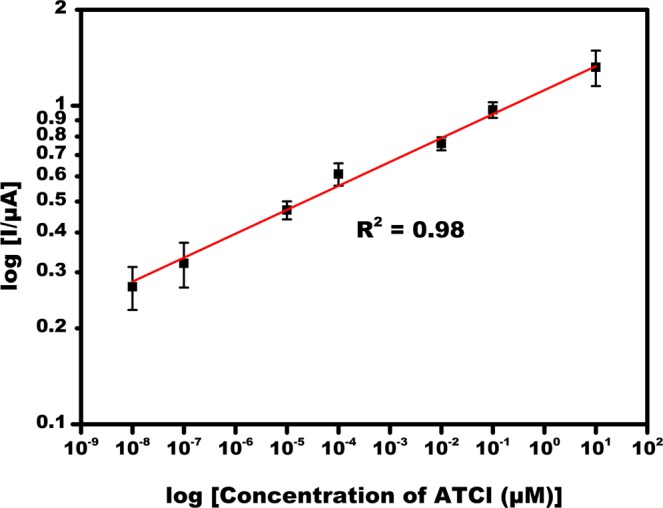
Calibration plot of log current Vs log concentration of substrate ATCl at different concentrations (10 fM, 100 fM, 10 pM, 100 pM, 10 nM, 100 nM and 10 µM).

At optimized conditions, DPV current was recorded at various substrate concentrations of ATCl, which helps to study the influence of substrate concentration to the AChE enzyme inhibition. In Fig. 5(d), due to the increase in concentration of ATCl ranging 10 fM to 10 µM in 0.1 M PBS containing 5 mM K [Fe(CN)_6_]^3−/4−^ the peak current also increased, which was a clear indication of ATCl oxidation due to the electrocatalytic reaction of the immobilized AChE enzyme and ATCl. The maximum current response was obtained at 10 µM substrate concentration, which showed the best enzyme activity and this concentration was used for subsequent experiments. The calibration plot of substrate concentration with linear regression equation 1/I_o_ = 0.08 (1/C_ATCl_) +0.52 and R^2^ = 0.98 follows Michaelis Menten kinetics (Fig. 6). Lineweaver-Burk equation (Eq. 1) was used to calculate Michaelis Menten constant (K_m_) and found to be 1.4 µM^13^.

1 $$1/{{\rm{I}}}_{{\rm{o}}}={{\rm{K}}}_{{\rm{m}}}/{{\rm{I}}}_{{\rm{\max }}}(1/{{\rm{C}}}_{{\rm{ATCl}}})+1/{{\rm{I}}}_{{\rm{\max }}}$$

### Analytical Performance of EµAD

**Figure 7:**
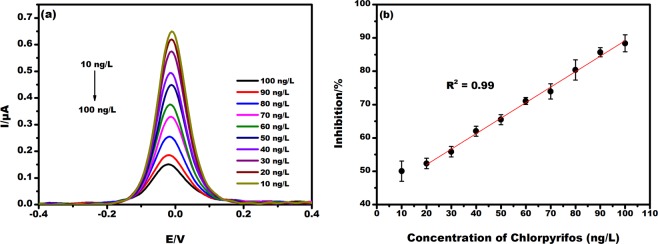
(**a**) DPV response of EµAD at various concentrations of chlorpyrifos ranging from 10 to 100 ng/L measured in 5 mM K [Fe(CN)_6_]^3−/4−^ and 10 µM ATCl prepared in 0.1 M PBS pH 7. (**b**) Standard curve of % Inhibition of EµAD Electrode versus concentration ranging from 10 to 100 ng/L in 5 mM K [Fe(CN)_6_]^3−/4−^ and 10 µM ATCl prepared in 0.1 M PBS pH 7.

EµAD’s response was measured in the presence and absence of chlorpyrifos solution. DPV response at different concentrations of chlorpyrifos ranging from 10 to 100 ng/L as shown in Fig. 7(a). The peak current of EµAD with and without chlorpyrifos was found to be 1.3 µA and 0.65 µA respectively. This sharp decrease in the current signifies blocking of active sites of the enzyme by chlorpyrifos, which inhibits the specific interaction of ATCl with an enzyme^32^. Figure 7(b) shows the standard curve of % Inhibition vs chlorpyrifos concentration. % inhibition values and concentration of chlorpyrifos were found to be directly proportional to each other within the linear concentration range^33^. The linear regression equation for DPV studies was formulated by the slope and intercept values from the standard calibration curve. Intercept and slope was calculated as 42.66 ± 0.76 and 0.47 ± 0.012 with equation % Inhibition = 0.47 × +42.66 (x = Concentration of chlorpyrifos (ng/L)) and R^2^ = 0.99 for the concentration ranging from 10 to 100 ng/L. The developed EµAD has the linear detection range of 10 to 100 ng/L and LoD calculated using 3σ rule was 6 ng/L^34^. The sensitivity of the sensor calculated by the formula Sensitivity = Slope of calibration plot (µA/ngL^−1^)/Active Surface Area (mm^2^) is 0.598 µA/ng L^−1^/mm^2,35^.

**Figure 8:**
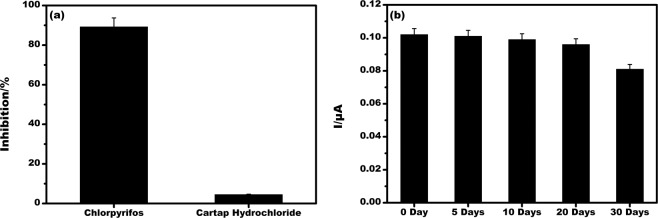
(**a**) Selectivity studies for EµAD at 100 ng/L of chlorpyrifos and cartap hydrochloride in 5 mM K [Fe(CN)_6_]^3−/4−^ and 10 µM ATCl prepared in 0.1 M PBS pH 7. **(b)** Stability studies of EµAD in 5 mM K [Fe(CN)_6_]^3−/4−^ and 10 µM ATCl prepared in 0.1 M PBS pH 7.

The biosensor was also tested with real vegetable samples (cucumber, capsicum, and brinjal) prepared as explained in the previous section and % recovery study for chlorpyrifos in the real vegetable sample was shown in Table 1. For the chlorpyrifos added to real vegetable samples, the recovery ratios were within the range 94 to 99% ± 4%, and RSD values less than 5%. From the data, it is evident that the EµAD showed good accuracy in testing real vegetable samples. In order to show the selectivity, the sensor was exposed to cartap hydrochloride a class of pesticide which is not having a phosphate group. 100 ng/L of cartap hydrochloride and chlorpyrifos was tested on the sensor and the % inhibition for chlorpyrifos and cartap hydrochloride was found to be 89.3% and 4.5% respectively, as shown in Fig. 8(a). It suggests the selective nature of the present sensor for chlorpyrifos specifically having a phosphate group. The permissible maximum residue limits (MRLs) of chlorpyrifos according to EU standards is 50 µg/L and the developed EµAD was able to detect a minimum value of 6 ng/L. The stability of the biosensor was tested up to 30 days, there was no major change in its response until 20 days and after that, there was a minimal decrease in current response which proved that the biosensor showed excellent stability till 20 days, Fig. 8(b).

**Table 1:** Recovery studies for chlorpyrifos added with the real vegetable sample by the present sensor.

Sample	Added (ng/L)	Found (ng/L)	% Recovery	%RSD
Cucumber	50	49.2	98.4	1.89
Capsicum	50	48.6	97.2	1.15
Brinjal	50	47.3	94.6	3.67

### Integration of EµAD with portable potentiostat

**Figure 9:**
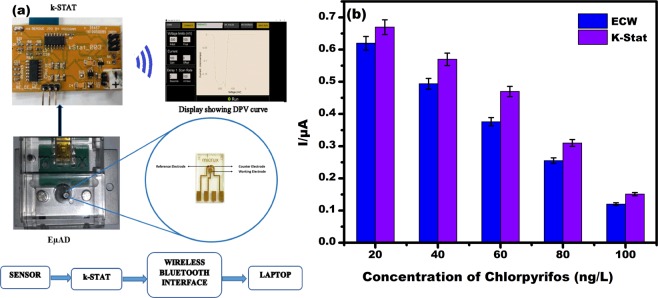
(**a**) Schematic representing EµAD interface with k-Stat, **(b)** Comparative bar graph depicting DPV peak current at different concentrations of chlorpyrifos (20, 40, 60, 80, 100 ng/L) as measured by k-Stat and conventional electrochemical workstation (ECW).

Figure 9(a) represents the schematic diagram of the integration of EµAD with as purchased portable potentiostat (k-Stat). k-Stat can measure various electrochemical parameters using CV and DPV techniques. Integration of EµAD with k-Stat gives the flexibility of using the proposed sensor in real time environment by a person with minimum scientific knowledge. k-Stat is low powered, portable potentiostat which needs 5 V DC for its operation. It also has the capability of transferring data wirelessly. Figure 9(b) shows comparative data obtained from EµAD attached with conventional electrochemical workstation (ECW) and a portable potentiostat (k-Stat), the comparative data is in a close match with each other. This integration of EµAD with the portable electronics gives the feasibility of using EµAD for real-time on-field analysis for the detection of chlorpyrifos OP in low concentration levels available in the food chain.

## Methods

### Reagents and apparatus

Acetylcholinesterase (AChE) from Electrophorus electricus (electric eel), Acetyl Thiocholine Chloride (ATCl), Pestenal Chlorpyrifos, Basolite Z1200 MOF were purchased from Sigma Aldrich. Sodium chloride (NaCl), potassium ferricyanide K_3_[Fe(CN)_6_], potassium ferrocyanide K_4_[Fe(CN)_6_], sodium dihydrogen orthophosphate (NaH_2_PO_4_) and Disodium hydrogen orthophosphate (Na2HPO4) was purchased from Fisher Scientific. Cartap Hydrochloride 50% w/w was bought from Dhanuka Agritech Ltd. All experiments were performed using Milli-Q deionized water having a resistance value of 18.2 MΩ and analytical grade chemicals.

The electrochemical analysis such as CV, DPV, EIS was done using potentiostat/galvanostat (Multiautolab (MA204), Autolab, The Netherlands). Thin film gold micro-electrodes (µE) having 10 mm height, 6 mm width, 0.75 mm thickness with 1 mm diameter working area and all-in-one cell platform facilitating a low reagent consumption (2 µL to 10 µL) were purchased from Micrux Technologies Ltd., Spain. k-Stat was purchased from Smoky Mountain Scientific, U.S.A. Fourier transform infrared (FTIR) spectroscopic analysis was performed using Frontier Perkin Elmer. Scanning Electron Microscopy (SEM) was done using SEM QUANTA 200 F.

The CV studies were done at scan rate 10 mV/s with applied potential from −0.4 to +0.4 V in 0.1 M phosphate buffer saline (PBS, pH7), containing 5 mM K [Fe(CN)_6_]^3−/4−^. DPV studies were carried out at a potential ranging from −0.4 to +0.4 V; amplitude, 0.05 V; pulse period, 0.05 s in 0.1 M phosphate buffer saline (PBS, pH7), containing 5 mM K [Fe(CN)_6_]^3−/4−^. The EIS measurements were performed in 0.1 M phosphate buffer (PBS, pH7), containing 5 mM K [Fe(CN)_6_]^3−/4−^ with AC frequency ranging from 100 Hz to 100 kHz. For measurements using k-Stat, the potential range from +0.1 V to −0.3 V was chosen with the scan rate of 100 mV/s in the presence of supporting electrolyte containing 5 mM K [Fe(CN)_6_]^3−/4−^ prepared in 0.1 M PBS, pH 7.

### Sensor performance

The electro-catalytic sensing performance was measured before and after incubating the biosensor in chlorpyrifos solution containing 0.1 M PBS pH 7, 5 mM K [Fe(CN)_6_]^3−/4−^ and 10 µM ATCl. The % Inhibition of chlorpyrifos to the enzyme was calculated from Eq. (2), where I_0_ and I_t_ represents the DPV peak current in response to 10 µM ATCl before and after the chlorpyrifos incubation respectively. The number of samples used were 20. The error bars are included in the calibration plot by taking standard deviation after repeating experiments for 3 times (n = 3). The average mean is plotted with standard error bars.

2 $$ \% \,{\rm{Inhibition}}=({{\rm{I}}}_{0}\,-\,{{\rm{I}}}_{{\rm{t}}})/{{\rm{I}}}_{0}\times 100 \% $$

### Sampling procedure for real samples

For a real vegetable sample analysis, 10 g of samples (cucumber, capsicum, and brinjal) was weighed and cut into small pieces. Then it was crushed finely using mortar and pestle following centrifugation at 3000 rpm for 15 min in PBS pH7. The supernatant was separated and known amount of chlorpyrifos was added for real vegetable sample analysis.

## Conclusion

This work demonstrated the use of bio-conjugated MOF with AChE enzyme grafted on the gold microelectrode on a microfluidic platform integrated with portable electronics for the selective detection of chlorpyrifos. This platform is a portable tool for on-site analyses. Implementation of mechanism assures robust mechanical transfer of a solid phase sample in to a reservoir for detection. The EµAD exhibited a good recognition capability toward chlorpyrifos, within the linear range from 10 to 100 ng/L with a low limit of detection of 6 ng/L with a sensitivity of 0.598 µA/ng L^−1^/mm^2^. The developed bio-sensor requires 2 µL of sample and remains stable for nearly a month. The integration of EµAD with portable potentiostat enables it to be used in collecting real-time data from on-field samples with minimum scientific skills. In summary these advantages render the novel EµAD platform an attractive candidate for on-site analysis. Further work is underway using another category of pesticides to make the setup more viable so that it can be used by farmers and others without technical knowledge.

## References

[1] Yadav IC (2015). Current status of persistent organic pesticides residues in air, water, and soil, and their possible effect on neighboring countries: A comprehensive review of India. Sci. Total Environ..

[2] Bajgar J (2004). Organophosphates/nerve agent poisoning: mechanism of action, diagnosis, prophylaxis, and treatment. Adv. Clin. Chem..

[3] Costa LG (2006). Current issues in organophosphate toxicology. Clin. Chim. Acta.

[4] Darvesh S (2008). Carbamates with Differential Mechanism of Inhibition Toward Acetylcholinesterase and Butyrylcholinesterase. J. Med. Chem..

[5] Grimalt S, Dehouck P (2016). Review of analytical methods for the determination of pesticide residues in grapes. J. Chromatogr. A.

[6] Virel A, Saa L, Pavlov V (2009). Modulated Growth of Nanoparticles. Application for Sensing Nerve Gases. Anal. Chem..

[7] Long Q, Li H, Zhang Y, Yao S (2015). Upconversion nanoparticle-based fluorescence resonance energy transfer assay for organophosphorus pesticides. Biosens. Bioelectron..

[8] Narakathu BB, Guo W, Obare SO, Atashbar MZ (2011). Sensors and Actuators B: Chemical Novel approach for detection of toxic organophosphorus compounds. Sensors Actuators B. Chem..

[9] Schulze H, Muench SB, Villatte F, Schmid RD, Bachmann TT (2005). Insecticide Detection through Protein Engineering of *Nippostrongylus b rasiliensis* Acetylcholinesterase B. Anal. Chem..

[10] Schöning MJ (2003). A dual amperometric/potentiometric FIA-based biosensor for the distinctive detection of organophosphorus pesticides. Sensors Actuators B Chem..

[11] Du D, Huang X, Cai J, Zhang A (2007). Amperometric detection of triazophos pesticide using acetylcholinesterase biosensor based on multiwall carbon nanotube–chitosan matrix. Sensors Actuators B Chem..

[12] Marrazza G (2014). Piezoelectric biosensors for organophosphate and carbamate pesticides: a review. Biosensors.

[13] Cui HF (2018). A highly stable acetylcholinesterase biosensor based on chitosan-TiO2-graphene nanocomposites for detection of organophosphate pesticides. Biosens. Bioelectron..

[14] Gadda G (2003). Kinetic mechanism of choline oxidase from Arthrobacter globiformis. Biochim. Biophys. Acta.

[15] Ghanem M, Fan F, Francis K, Gadda G (2003). Spectroscopic and kinetic properties of recombinant choline oxidase from Arthrobacter globiformis. Biochemistry.

[16] Chauhan N, Pundir CS (2012). An amperometric acetylcholinesterase sensor based on Fe3O4nanoparticle/multi-walled carbon nanotube-modified ITO-coated glass plate for the detection of pesticides. Electrochim. Acta.

[17] Talan A (2018). Ultrasensitive electrochemical immuno-sensing platform based on gold nanoparticles triggering chlorpyrifos detection in fruits and vegetables. Biosens. Bioelectron..

[18] Du D, Ding J, Cai J, Zhang J, Liu L (2008). *In situ* electrodeposited nanoparticles for facilitating electron transfer across self-assembled monolayers in biosensor design. Talanta.

[19] Govindasamy M, Mani V, Chen S-M, Chen T-W, Sundramoorthy AK (2017). Methyl parathion detection in vegetables and fruits using silver@graphene nanoribbons nanocomposite modified screen printed electrode. Sci. Rep..

[20] Chikae M (2006). Direct fabrication of catalytic metal nanoparticles onto the surface of a screen-printed carbon electrode. Electrochem. commun..

[21] Nesakumar N, Sethuraman S, Krishnan UM, Rayappan JBB (2016). Electrochemical acetylcholinesterase biosensor based on ZnO nanocuboids modified platinum electrode for the detection of carbosulfan in rice. Biosens. Bioelectron..

[22] Santos CS, Mossanha R, Pessôa CA (2015). Biosensor for carbaryl based on gold modified with PAMAM-G4 dendrimer. J. Appl. Electrochem..

[23] Chen D, Sun X, Guo Y, Qiao L, Wang X (2015). Acetylcholinesterase biosensor based on multi-walled carbon nanotubes-SnO2-chitosan nanocomposite. Bioprocess Biosyst. Eng..

[24] Deep A, Bhardwaj SK, Paul AK, Kim KH, Kumar P (2015). Surface assembly of nano-metal organic framework on amine functionalized indium tin oxide substrate for impedimetric sensing of parathion. Biosens. Bioelectron..

[25] Czaja AU, Trukhan N, Müller U (2009). Industrial applications of metal–organic frameworks. Chem. Soc. Rev..

[26] Cui Y, Yue Y, Qian G, Chen B (2012). Luminescent Functional Metal–Organic Frameworks. Chem. Rev..

[27] Li Y (2019). Co-MOF nanosheet array: A high-performance electrochemical sensor for non-enzymatic glucose detection. Sensors Actuators B Chem..

[28] Kumar P, Bansal V, Deep A, Kim K-H (2015). Synthesis and energy applications of metal organic frameworks. J. Porous Mater..

[29] Kumar Pawan, Deep Akash, Kim Ki-Hyun (2015). Metal organic frameworks for sensing applications. TrAC Trends in Analytical Chemistry.

[30] Li P (2016). Encapsulation of a Nerve Agent Detoxifying Enzyme by a Mesoporous Zirconium Metal–Organic Framework Engenders Thermal and Long-Term Stability. J. Am. Chem. Soc..

[31] Sahari SK, Sing JCH, Hamid KA (2009). The Effects of RCA Clean Variables on Particle Removal Efficiency. World Acad. Sci. Eng. Technol..

[32] Coban A, Carr RL, Chambers HW, Willeford KO, Chambers JE (2016). Comparison of inhibition kinetics of several organophosphates, including some nerve agent surrogates, using human erythrocyte and rat and mouse brain acetylcholinesterase. Toxicol. Lett..

[33] Zhang S, Zhao H, John R (2001). Development of a quantitative relationship between inhibition percentage and both incubation time and inhibitor concentration for inhibition biosensors—theoretical and practical considerations. Biosens. Bioelectron..

[34] Shrivastava A, Gupta V (2011). Methods for the determination of limit of detection and limit of quantitation of the analytical methods. Chronicles Young Sci..

[35] Balakrishnan SR (2015). A Point-of-Care Immunosensor for Human Chorionic Gonadotropin in Clinical Urine Samples Using a Cuneated Polysilicon Nanogap Lab-on-Chip. PLoS One.

